# 9.Q. Skills building seminar: Redistribution of ill-defined deaths: a methodological conundrum

**DOI:** 10.1093/eurpub/ckac129.618

**Published:** 2022-10-25

**Authors:** Organised by: COST Action CA18218– European Burden of Disease Network, Chair persons: Brecht Devleesschauwer (Belgium)

## Abstract

Today, several countries are conducting national burden of disease (BOD) studies, calculating Disability-Adjusted Life Years (DALYs) for a variety of diseases, injuries and risk factors. The DALY metric is composed of a morbidity component, the Years Lived with Disability (YLDs), and a mortality component, the Years of Life Lost (YLLs). The calculation of YLLs typically starts from national cause of death data, which are coded using the International Statistical Classification of Diseases and Related Health Problems (ICD). National BOD studies however often follow (a modified version of) the cause hierarchy of the Global Burden of Disease (GBD) study, which applies coarser categories, and does not contain unknown or ill-defined causes. This implies that not all ICD codes can be mapped to one specific BOD cause, giving rise to so-called “ill-defined” or “garbage” codes. To include all deaths in a national BOD study, it is therefore necessary to redistribute the ill-defined deaths to specific BOD causes. Several methodological approaches exist for redistribution of ill-defined deaths, ranging from the use of fixed redistribution proportions to advanced redistribution algorithms based on multiple cause of death data. The choice of the methodology needs to depend on the nature of the available cause of death data, and as a consequence, methodologies are typically tailored to the specific data at hand. The absence of a single best methodology, and the methodological heterogeneity across applications, has made redistribution of ill-defined deaths into a methodological conundrum. This skills building workshop will present the redistribution methods applied in different national burden of disease studies, including a discussion of their strengths and weaknesses. By providing a step-by-step presentation of how the methods have been applied, attendees will gain unique insights in the different redistribution methods currently applied.

**Key messages:**

• Redistribution of ill-defined deaths is an essential part of national burden of disease studies, but is methodologically challenging.

• Attendees will receive an overview of the redistribution methods applied in different national burden of disease studies, including a discussion of their strengths and weaknesses.

